# Ion and metabolite transport in the chloroplast of algae: lessons from land plants

**DOI:** 10.1007/s00018-018-2793-0

**Published:** 2018-03-14

**Authors:** Justine Marchand, Parisa Heydarizadeh, Benoît Schoefs, Cornelia Spetea

**Affiliations:** 1 Metabolism, Bioengineering of Microalgal Molecules and Applications (MIMMA), Mer Molécules Santé, IUML, FR 3473 CNRS, Le Mans University, 72000 Le Mans, France; 2 Department of Biological and Environmental Sciences, University of Gothenburg, 40530 Göteborg, Sweden

**Keywords:** Algae, Channel, Chloroplast, Metabolism, Photosynthesis, Transporter

## Abstract

Chloroplasts are endosymbiotic organelles and play crucial roles in energy supply and metabolism of eukaryotic photosynthetic organisms (algae and land plants). They harbor channels and transporters in the envelope and thylakoid membranes, mediating the exchange of ions and metabolites with the cytosol and the chloroplast stroma and between the different chloroplast subcompartments. In secondarily evolved algae, three or four envelope membranes surround the chloroplast, making more complex the exchange of ions and metabolites. Despite the importance of transport proteins for the optimal functioning of the chloroplast in algae, and that many land plant homologues have been predicted, experimental evidence and molecular characterization are missing in most cases. Here, we provide an overview of the current knowledge about ion and metabolite transport in the chloroplast from algae. The main aspects reviewed are localization and activity of the transport proteins from algae and/or of homologues from other organisms including land plants. Most chloroplast transporters were identified in the green alga *Chlamydomonas reinhardtii*, reside in the envelope and participate in carbon acquisition and metabolism. Only a few identified algal transporters are located in the thylakoid membrane and play role in ion transport. The presence of genes for putative transporters in green algae, red algae, diatoms, glaucophytes and cryptophytes is discussed, and roles in the chloroplast are suggested. A deep knowledge in this field is required because algae represent a potential source of biomass and valuable metabolites for industry, medicine and agriculture.

**Electronic supplementary material:**

The online version of this article (10.1007/s00018-018-2793-0) contains supplementary material, which is available to authorized users.

## Introduction

Cellular metabolism consists of chains of reactions converting substrates to products with the help of enzymes, energy and reductants. The implementation of the metabolism requires fluxes of compounds that are in first approximation controlled by both enzyme kinetics and compound diffusion. Diffusion of compounds can be considered at the microscopic or macroscopic scales. At microscopic scale, the channeling of substrates and products takes place from one enzyme to another without releasing them in the bulk phase [1]. The macroscopic scale is represented by the physical transfer of substrates and products through a barrier. The requirement of this kind of transport originates from the compartmentalization of many biochemical activities. For example, the production of ATP in the chloroplast depends on the existence of a proton (H^+^) gradient that itself cannot be built without the existence of two compartments separated by a membrane. The complexity of cell compartmentalization, which already existed in the prokaryotic cyanobacteria, increased dramatically with the prokaryotic-to-eukaryotic transition and the evolution of the different branches of the algal phyla. Beyond the purely scientific interest in ion and metabolite transport for the understanding of algal physiology, a deep knowledge in this field is important, because algae represent a potential platform for the production of commercially interesting compounds [2–4]. In the development of biotechnological methodologies non-destructive to the biomass [2], a deep knowledge on transporters residing in the different compartments (e.g., [5]) will constitute a great advantage.

### The success of endosymbiotic organisms relies on trading between cell compartments: the absolute requirement for transport proteins

The chloroplast is an organelle specific for eukaryotic oxygenic photosynthetic organisms, namely algae and land plants. It is mostly known for being the host of a fundamental process called photosynthesis, which generates molecular oxygen and organic molecules in the Earth’s biosphere since billion years ago. In addition, it is the most prominent member of the plastid family of organelles, involved in many biosynthetic pathways such as those for production of carotenoids, lipids, amino acids, phytohormones, etc. [6–8]. The chloroplast is a heavily compartmented organelle: the outer and the inner envelope surrounding the organelle, the soluble stroma, the thylakoid membrane, and the enclosed (lumenal) space.

How chloroplasts appeared during evolution remains a tremendously exciting question. Several hypotheses have been proposed. The strongest, and so far adopted one, is that proposed by Margulis [9], according to which ancestral anaerobic eukaryotes became able to ingest solid particles such as photosynthetic prokaryotes related to cyanobacteria. In some cases, the ingested cyanobacteria continued to live and eventually evolved into chloroplasts surrounded by two membranes (primary plastids). The fact that algal and land plant cells take benefits from the energy-rich carbohydrates produced by the chloroplast leads Mereschkowsky in 1905 to postulate that the exchange of carbohydrates might have been crucial for the stability of the host–endosymbiont relationship [10]. However, a membrane represents an insurmountable barrier for hydrophilic compounds such as carbohydrates. The crossing is even more difficult when the chloroplast is surrounded by three or four membranes (secondary plastids) [6]. Therefore, to gain access to the molecules produced by the endosymbiont, crossing of membranes should be facilitated [11]. The facilitators are membrane-spanning transport proteins. Today, the chloroplast membranes (envelope and thylakoid) can be seen as selective filters across which different types of compounds such as small ions, metabolites, and nucleotides are transported through channels, secondary transporters and primary transporters/pumps, classified according to the Transport Classification System [12].

It is clearly established that the good fitness of a photosynthetic cell relies on chloroplast activity. Chloroplasts are sensitive to changes in the intensity of the environmental constraints [13] and have developed strategies to survive the stress. Many of these strategies require changes in the nuclear gene expression, which are mediated by chloroplast signals sent to the nucleus (retrograde signalling, [14]). According to the current knowledge, retrograde signalling would involve, but not exclusively, metabolites [15], sugar [16], chlorophyll-precursors [17–19], ions [20], reactive oxygen species [21], chloroplast-encoded proteins [22] and carotenoid-derivatives [23, 24] to be transported across chloroplast membranes.

It is evident that gene loss and lateral gene transfer have played an important role in the evolution of unicellular eukaryotic algae, as witnessed by comparative genomic studies of diatoms and red algae [25]. These have resulted in modification of the transporter repertoire and/or their activity to satisfy the cell metabolic demands. A deeper knowledge in transporters of algae is required for the reconstruction of genome-scale metabolic networks and prediction of metabolic fluxes in ‘control’ environment as well as for studying the impact of mutations or changes in the environmental constraints on metabolic fluxes [26–28].

### Algal phylogeny and models

**Fig. 1:**
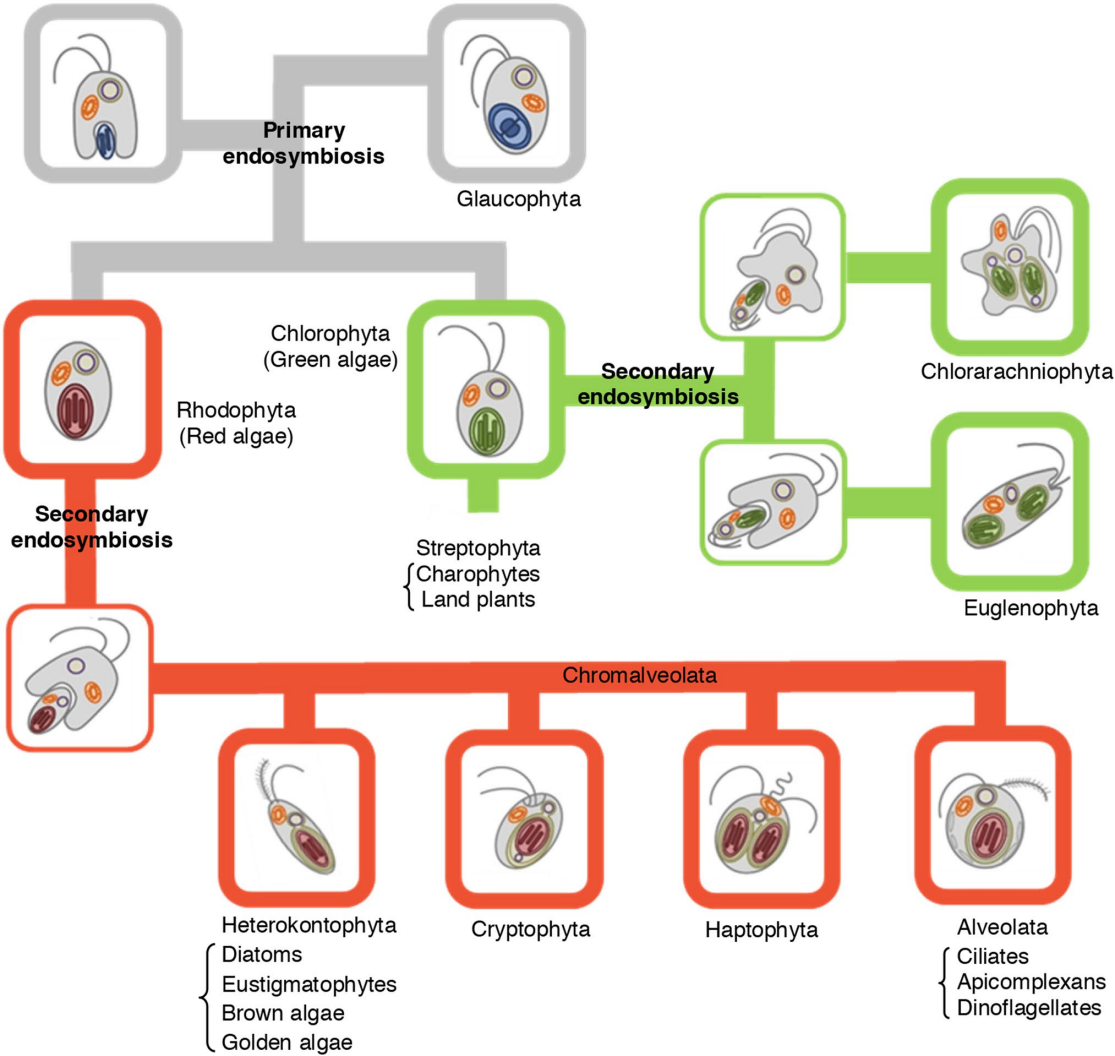
Schematic view of plastid evolution in the history of photosynthetic eukaryotes. The engulfment of a cyanobacterium by a primitive eukaryotic host (primary endosymbiosis) gave rise to three algal lineages: Chlorophyta, Rhodophyta and Glaucophyta. The green lineage Streptophyta evolved from Chlorophyta. The engulfment of green and red algae by different hosts (secondary endosymbiosis) resulted in the green lineages Euglenophyta and Chlorarachniophyta and in the red lineage Chromalveolata, that is further divided into four major subgroups, namely Heterokontophyta, Cryptophyta, Haptophyta and Alveolata. Primary plastids are surrounded by two membranes, whereas three or four membranes can be present in the secondary plastids. The figure is a modified version of Fig. 1 from Facchinelli and Weber [30] (reproduced with permission from the authors)

Plastids and algal evolution are tightly linked as briefly explained below. The primary endosymbiosis resulted in the so-called ‘proto-alga’, a unique alga with two membranes surrounding the plastid, that diverged into the green and red algal lineages as well as to the glaucophytes, all characterized by different plastid architectures (Fig. 1). Land plants arose following the evolution of multicellularity within the green algal lineage and have the charophytes as the closest relatives (together known as the streptophytes). All these eukaryotes (the land plants, the green algae, the red algae, and the glaucophytes) having plastids surrounded by two membranes comprise the so-called Archaeplastida group. Subsequent secondary endosymbiosis of green algae or red algae engulfed by different non-photosynthetic eukaryotic hosts resulted in the euglenophytes and chlorarachniophytes (having chloroplasts with three membranes) and the chromalveolates (having chloroplasts with four membranes), respectively (Fig. 1; [6, 29]). It is believed that a single event is at the origin of all chromalveolates, which are subdivided in heterokonts (diatoms, eustigmatophytes, golden algae and brown algae), haptophytes, cryptophytes, and alveolates (ciliates, apicomplexans and dinoflagellates) [30–32]. Some chromalveolates also share common origin with rhizarians [33]. However, the number of secondary endosymbiotic events is still under debate [32, 34]. The evolutionary history could explain the many similarities at the biochemistry and cell physiology levels in algae and land plants, and also why they diverged enormously in many other aspects. For instance, diatoms have a urea cycle derived from the eukaryotic host [35] and an Entner–Doudoroff pathway in mitochondria, reminiscent of glycolysis in prokaryotes [36]. Furthermore, in land plants glycolysis takes place entirely in the cytosol, whereas in green algae the upper half is localized in the chloroplast [37].

**Fig. 2:**
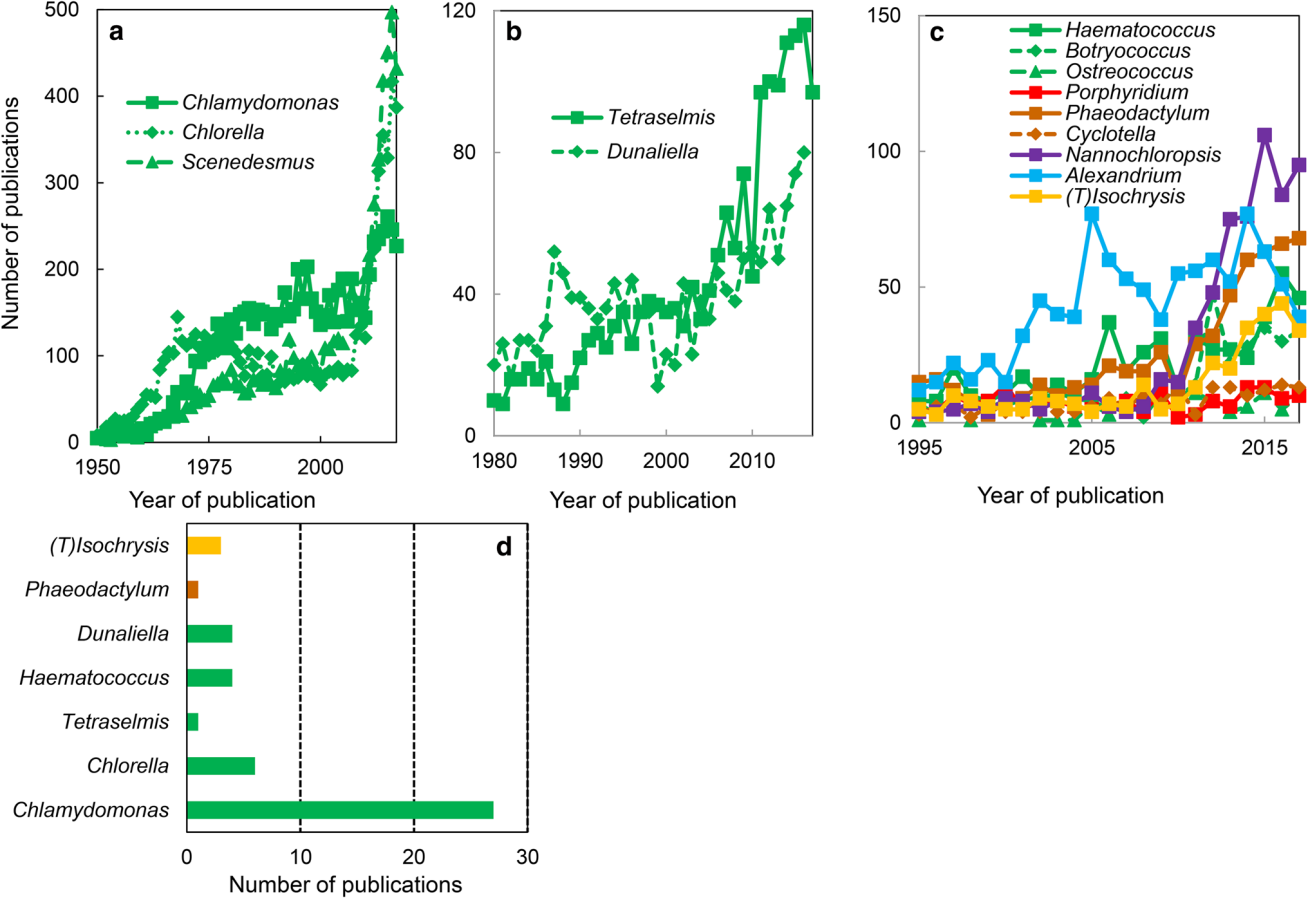
Algal models. Data mining in ‘all databases’ of the Web of Science using ‘algal genus’ (**a**–**c**) or ‘algal genus and transporter’ (**d**) as keywords in titles of publications. The data revealed three categories, namely historical, recent and emerging models (see the text for details). The most used models for transporter studies belong to the green algae and include *Chlamydomonas*. The colors used to represent various algal groups were green (green algae), red (red algae), brown (diatoms), magenta (eustigmatophytes), blue (alveolates) and yellow (haptophytes)

Model organisms are taxons that have been studied because they present particular experimental advantages and are easy to maintain in the laboratory [38]. Data mining in Web of Science (https://clarivate.com/products/web-of-science/) using ‘algal genus’ as keyword in titles of publications revealed three categories of algal models (Fig. 2). In the first category are ranked the historical models, namely those that emerged around 1950 and have been widely used since the beginning of the 20th century. This category contains three green algal genera, namely *Chlamydomonas, Chlorella,* and *Scenedesmus* (Fig. 2a). They correspond to the period when the mechanism of photosynthesis was investigated, and algae represented a system mimicking photosynthetic leaf cells, but much easier to manipulate while maintaining the activity in vivo (for a review, see [39]). The second category contains the recent models, namely taxons that were used starting 1980. Surprisingly, this category contains two additional green algal genera, namely *Tetraselmis* and *Dunaliella* (Fig. 2b). This period corresponds to the introduction of genome transformation methods in algae (e.g., [40]) and to the use of algae as sources of biomass and metabolites [41–43]. The last category consists of the models emerging since the start of the 20th century (Fig. 2c). The genomes of taxons belonging to these genera have been sequenced (for an updated list, see https://en.wikipedia.org/wiki/List_of_sequenced_plant_genomes), providing a unique set of data for molecular, biochemical, and physiological research (e.g., [28]). When using the same strategy in Web of Science to find the algal models employed to study transporters, the most investigated genus was the one of *Chlamydomonas* (Fig. 2d).

### The chloroplast: a highly compartmentalized organelle

Many biosynthetic pathways in the chloroplast require soluble and membrane-attached proteins. In addition, the photobiochemical pathways of photosynthesis (also known as the light-dependent reactions) rely on a complex membrane architecture taking its origin in the cyanobacterial ancestor. Already in these ‘primitive’ organisms, the light-dependent reactions take place in sheet-like membranes called thylakoids, which are in direct contact with the cytosol. In algae and land plants, thylakoids are separated from the cytosol by chloroplast envelope membranes. Glaucophytes and also the rhizopod *Paulinella chromatophora* were believed to occupy an intermediate position between cyanobacteria and chloroplasts, because their photosynthetic apparatus, the so-called cyanelle, is surrounded by a peptidoglycan layer that is believed to be a relic of the endosymbiotic origin of chloroplasts from cyanobacteria. Recently, the presence of a peptidoglycan between the inner and the outer envelope membranes has been traced until the moss chloroplast [44–46]. Homologues of genes required for peptidoglycan synthesis have been found in the genome of the charophyte *Klebsormidium nitens* (syn. *K. flaccidum*) [47], the green alga *Micromonas* sp. CCMP 1545 [48], moss [49], lycophytes [50], gymnosperms [51] and angiosperms such as *Arabidopsis thaliana* (*A. thaliana*) [52]. In the moss, the role of the peptidoglycan is related—at least—to chloroplast division. In gymnosperms and *A. thaliana*, it seems that the role of peptidoglycan in chloroplast division has been lost, but it remains essential in chloroplast development [52].

Much of our understanding of thylakoid architecture comes from transmission electron microscopy studies (green algae: [53], diatoms: [54, 55], red algae: [56]). These studies revealed that the organization of thylakoid membranes differs among the algal lineages. While thylakoids of green algae arrange similar to those of land plants and comprise grana stacks and intergranal thylakoids, those of diatoms arrange usually as groups of three, and thylakoids of red algae remain unstacked because of the presence of non-membranous light-harvesting antennas (phycobilisomes). It is out of scope of this review to provide the reader with a full description of the plastid architecture (for review, see [6]). Nevertheless, the theme of this review needs to emphasize the number of subcompartments of a chloroplast: outer plastid membrane, inner envelope membrane, inter-envelope space, stroma, grana, thylakoid lumen and intergranal (stroma) thylakoids. An in situ cryo-electron tomographic study of the green alga *Chlamydomonas reinhardtii* (*C. reinhardtii*) has revealed that the distinction between grana and intergranal thylakoid is not so obvious as it is in land plants [57].

In algal phyla that have emerged as a result of secondary endosymbiotic events, the chloroplast has three-to-four membranes [29, 34, 58], resembling a matryoshka. A typical example of complex plastid architecture is the case of diatoms, in which the outer envelope is surrounded by two additional membranes [59]. The outermost membrane is the chloroplast endoplasmic reticulum (cER) [60], a membrane that is supposed to derive from the host phagocytic vacuole, though this particular point is still debated [61]. Underneath is the periplastidial membrane (PPM), considered as a relic of the host plasma membrane [62]. The two innermost membranes correspond to the two plastid envelopes described above: the outer plastid membrane (OPM, another host-derived membrane), and the inner envelope membrane (IEM, that has an endosymbiont origin). The space between the PPM and the OPM, also called the periplastid compartment (PPC), corresponds to the symbiont cytoplasm. In some groups such as cryptophytes, a reduced version of the symbiont nucleus remains, whereas in diatoms, all the genes have been transferred [63]. A trivial consequence of the presence of these additional membranes would be the requirement for additional transport proteins mediating translocation of compounds between the different plastid subcompartments. In diatoms, the cER is directly connected to the host outer nuclear envelope and ER [32], whereas in some brown algae, the endomembrane system is not continuous, and transfers the material through a vesicular network [64]. Recently, a very comprehensive study has revealed the presence of a vesicular network within the PPC [65], therefore, unifying the presence of a vesicular network to facilitate the transport between the nucleus and the chloroplast.

### Photobiochemical reactions and CO_2_ fixation: the need for transporters

The photobiochemical reactions involve four macrocomplexes in thylakoids, namely photosystem II (PSII), photosystem I (PSI), the cytochrome *b*_6_*f* complex, and the H^+^-translocating ATP synthase (CF_0_F_1_). A brief description of these reactions is provided here to emphasize the need for transporters in the thylakoid membrane. The reader interested in more details on the functioning of the photobiochemistry of photosynthesis is referred to [66].

The energy associated with photons absorbed by the pigment bed forming the thylakoid-located light-harvesting complexes of PSII and PSI is used to drive electron transfer reactions resulting in NADPH in the stroma. Water oxidation in the thylakoid lumen generates molecular oxygen and electrons that simultaneously trigger H^+^ accumulation in this compartment. The resulting H^+^ gradient across the thylakoid membrane (pmf) is used by the ATP synthase to generate ATP in the stroma, and also to drive the exchange of ions against their electrochemical gradient. The pmf is composed of an electric field and a pH gradient (ΔΨ and ΔpH, respectively).

The water oxidation reactions require water, a cluster of 4 Mn ions, and the presence of Ca^2+^ and Cl^−^ for optimal function [67]. The delivery modes of these ions and water to the lumen remain largely uncharacterized [68]. The electron transport chain is composed of fixed and mobile electron carriers, such as plastocyanin (PC, a Cu^+^–protein complex) located in the thylakoid lumen. On the other hand, the ‘manipulation’ in an environment of oxygen, light, and electrons increases the oxidative risk. To fight against this risk, the chloroplast is equipped with several mechanisms for dissipation of excess light [69] and antioxidative metalloenzymes such as superoxide dismutases [70]. Despite the importance of these proteins for the functioning of the chloroplast and photosynthesis, the import pathways of their metallic counterpart (Cu^+^, Zn^2+^ and Fe^2+^) into the chloroplast stroma and thylakoid lumen remain largely unknown.

The ATP and NADPH produced by the photobiochemical reactions of photosynthesis in thylakoids are used in the CO_2_ fixation (Calvin–Benson) cycle. In C3 plants, this cycle starts with the incorporation of CO_2_ into ribulose-5-bisphosphate (RuBP) resulting into two glyceraldehyde 3-phosphate (G3P) molecules. One of the G3P molecules is used for the synthesis of more complex molecules, whereas the second one is used to regenerate the pool of RuBP. The RuBP carboxylase/oxygenase (RuBisCO) is the key enzyme of the cycle that can use either CO_2_ or O_2_ as substrates depending on their ratio in its close environment. In C4 plants [71], green algae, and diatoms [72], CO_2_ is first incorporated in phosphoenolpyruvate (PEP) resulting in oxaloacetate (OAA), which is converted to malate in cytosol, and then transported to the chloroplast for decarboxylation, delivering CO_2_ to RuBisCO. This mechanism known as the biochemical CO_2_ concentration mechanism (CCM) allows achieving significant CO_2_ levels in the proximity of RuBisCO and competes out photorespiration.

In contrast to land plants that have an easy access to atmospheric gaseous CO_2_, algae live in aqueous environments where CO_2_ is available as dissolved inorganic carbon (Ci) species (CO_2_, HCO_3_^−^ and CO_3_^2−^) at levels varying over time and space due to the following factors: (1) sediment or soil respiration; (2) pH affecting the balance between CO_2_ and carbonic acid species; and (3) slower CO_2_ diffusion in aquatic environments (10^4^ fold slower in water than in the air). These factors lead to the possibility of enhanced oxygenase activity of RuBisCO, resulting in photorespiration at the expense of carbon fixation. To maintain as high as possible carbon fixation activity, algal RuBisCO is concentrated in the pyrenoid (diatoms: [73]; green algae: [74–76]), and fed with CO_2_ thanks to the catalytic activity of carbonic anhydrases (CAHs) [77]. To ensure feeding of CAHs with CO_2_, algae have at disposal active Ci uptake mechanisms known as biophysical CCMs [78–80]. The CCMs as well as the exchange of carbohydrates and other metabolites between the chloroplast and the cytosol require specialized transport proteins, whose identities in most algal groups are not known.

**Fig. 3:**
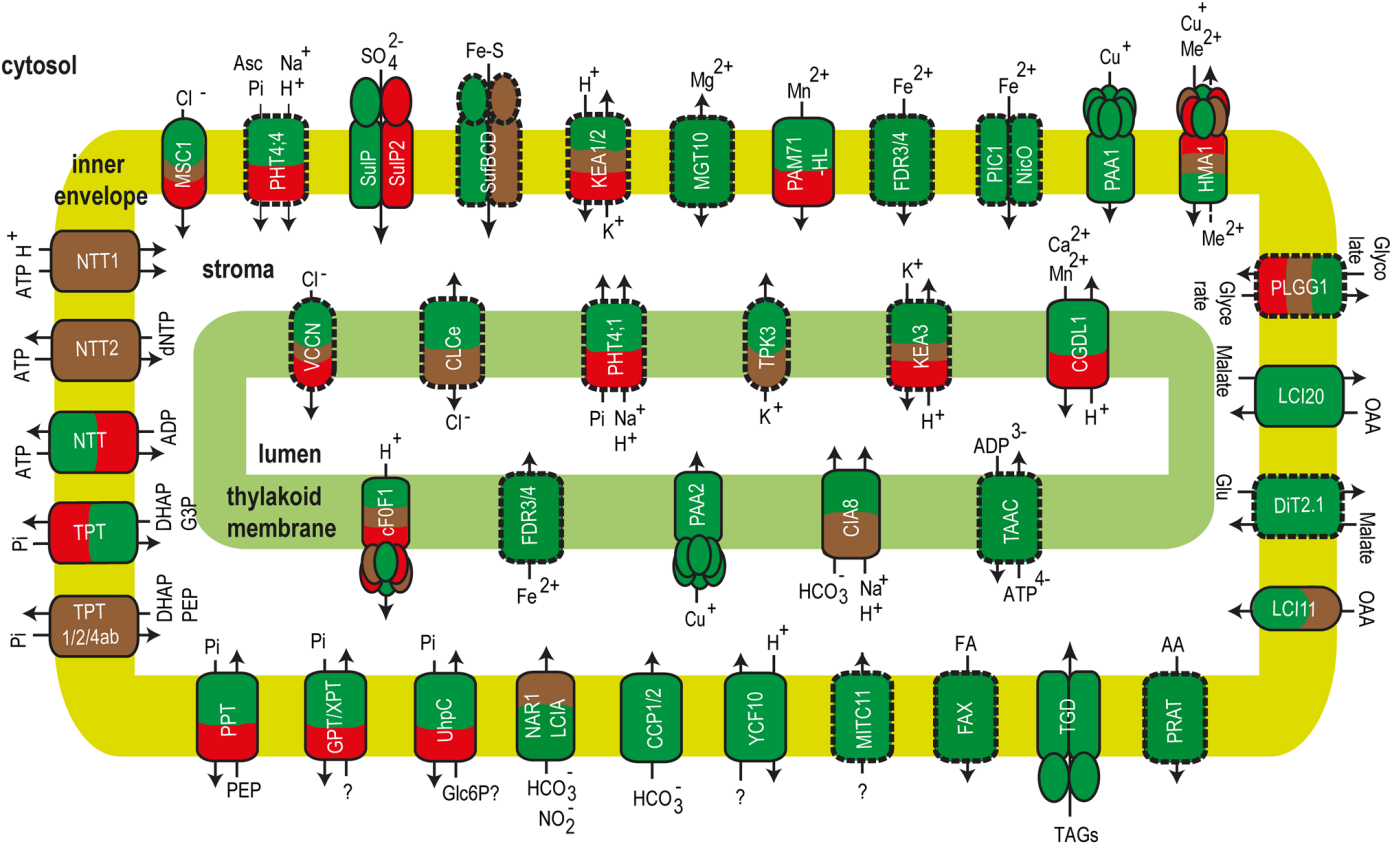
Ion and metabolite transport proteins of the chloroplast envelope and thylakoid membrane from algae. Proteins identified or characterized in at least one algal model are framed with continuous line. Plant homologues genes coding for putative transporters in algae are framed with broken lines. Green algae, red algae and diatoms are represented in green, red and brown, respectively

Comprehensive reviews and a Research Topic have been recently dedicated to ion and metabolite transport in the chloroplast of land plants [81–86]. In this review, we present the current knowledge about transport of ions and metabolites in algal chloroplasts. Supplemental Table S1 includes information about identified/characterized transport proteins and the presence of homologues genes to those characterized in land plants (*A. thaliana, Zea mays*) in algal models for green algae: *C. reinhardtii* and *Volvox carteri* (*V. carteri*), red algae: *Galdieria sulphuraria* (*G. sulphuraria*) and *Cyanidioschyzon merolae* (*C. merolae*), diatoms: *Thalassiosira pseudonana* (*T. pseudonana*) and *Phaeodactylum tricornutum* (*P. tricornutum*), glaucophytes: *Cyanophora paradoxa* (*C. paradoxa*) and cryptophytes: *Guillardia theta* (*G. theta*). Figure 3 provides an overview of algal transport proteins in green algae, red algae and diatoms.

## Chloroplast ion transport

Ions are involved in many biosynthetic pathways and physiological processes in all types of organisms and cells. Ions are critical for the pH, volume, and osmoregulation of intracellular compartments as well as for intercellular communication. When present in non-adequate amounts, ions may cause stress-related symptoms (e.g., [87]). Ions have to be imported from the cytosol to reach the various plastid subcompartments of algae. Because of the charged nature of ions, they cannot spontaneously cross membranes and need to be translocated by ion channels, secondary transporters and pumps. Channels transport ions down their concentration gradient without consuming ATP. Pumps need ATP to transport ions across membranes, whereas secondary transporters (symporters and antiporters) use the concentration gradient of a co-transported ion.

Main anions used in the chloroplast are chloride (Cl^−^: pmf regulation, osmosis), phosphate (Pi: ATP formation), sulfate (SO_4_^2−^: formation of cysteine and methionine), nitrite (NO_2_^−^: nitrogen assimilation) and bicarbonate (HCO_3_^−^: carbon assimilation) [88]. Most abundant cation in the chloroplast is potassium (K^+^), used in osmosis and pmf regulation, but divalent cations such as calcium, magnesium, manganese and iron (Ca^2+^, Mg^2+^, Mn^2+^ and Fe^2+^) are also present, since they are required as cofactors for enzymes, signaling, water oxidation, electron transport and thylakoid organization [88]. Despite multiple evidence for ion–dependent activities in the chloroplast, the identity of the dedicated transport proteins is in most cases missing in algae and even in land plants [89, 90].

### Chloride transport

Evidence for the existence of voltage-dependent anion channel activities has been reported for thylakoid membranes from the giant charophyte alga *Nitellopsis obtuse* [91] and also from land plants [92, 93]. It was only in the recent years that genes have been annotated with functions in such activities. In *A. thaliana* thylakoids, two types of Cl^−^ channels have been characterized, namely the CLCe member of the CLC family [94, 95], and the VCCN member of a new family of voltage-dependent chloride channels (VCCN1 and VCCN2; [96, 97]). Phenotypic characterization of mutants lacking either CLCe or VCCN1 suggested altered Cl^−^ homeostasis and enhanced pmf partitioning to ΔΨ at the expense of ΔpH across the thylakoid membrane [95–97]. Since light energy distribution between electron transport and photoprotective dissipation of excess light (NPQ) are sensitive to changes in pmf composition, the *vccn1* mutants displayed slower NPQ induction when exposed to repetitive sudden transitions from low-to-high light [96, 97]. Altered Cl^−^ homeostasis by the two proteins influenced thylakoid ultrastructure, macroorganization of PSII supercomplexes as well as state transitions in *A. thaliana* [95, 96]. To cope with fluctuations in their environment, algae also adjust photosynthesis using pH-dependent NPQ and state transition mechanisms ([69], for a review, see [98]). Phylogenetic analyses revealed candidates for CLCe-like genes in green algae, diatoms and cryptophytes [90], whereas candidates for *VCCN*-like genes were found in green algae, red algae, diatoms and cryptophytes [96]. It remains to be studied whether VCCN1- and CLCe-like proteins transport Cl^−^ or other anions in algae, and if they are involved in regulation of photosynthesis or other processes in the chloroplast.

Adaptation to osmotic shock in living organisms relies on the presence of mechanosensitive ion channels (MscS; [99]). All analysed photosynthetic eukaryotes have at least one MscS-like (MSL) sequence [100]. The chloroplast envelope of *A. thaliana* harbors two envelope-located MSL proteins (MSL2 and MSL3), which were shown to play roles in efflux of osmolytic ions (Na^+^, Cl^−^, H^+^ and Ca^2+^) affecting chloroplast shape and size [101]. They are activated by increased membrane tension, which can happen during normal growth and development as well as under osmotic stress. One mechanosensitive ion channel named MSC1 was identified in *C. reinhardtii* as a homologue of *Escherichia coli* (*E. coli*) MscS and was localized to the chloroplast [102]. The MSC1 channel displayed a strong preference for anions over cations, feature that makes it distinct from the plant MSLs. The Cl^−^ ion is proposed as the most likely substrate, and to have a function in maintaining optimal chloroplast organization in *C. reinhardtii* [102].

### Phosphate transport

Transporters driving the net flow of Pi into the chloroplast belong to the PHT2 and PHT4 families. They have been intensively studied in *A. thaliana,* and include members localized to either the envelope (PHT2;1: [103], PHT4;2, PHT4;4 and PHT4;5: [104]) or the thylakoid membrane (PHT4;1: [105]). Yeast complementation experiments revealed a H^+^-dependent mechanism for Pi transport by both PHT2 and PHT4 members [103, 104]; however, transport assays in *E. coli* indicated a Na^+^-dependent mechanism for Pi transport by the thylakoid PHT4;1 [105, 106]. More recently, the envelope PHT4;4 transporter was found to function in ascorbate rather than Pi supply into the stroma [107]. PHT4;1 was proposed as a local Pi supplier to the ATP synthase in thylakoids during the photobiochemical reactions [108]. PHT2 family is represented in land plants and charophytes, but not in unicellular green algae or any other algae groups [109]. Phylogenetic analyses of PHT4 revealed homologues sequences in green algae and red algae but not in other groups [90]. Most algae harbor plastidic Pi transporters that work in exchange with phosphorylated organic compounds (see “Carbohydrate transport”).

### Sulfur transport

Sulfate is transported into the chloroplast stroma of *A. thaliana* via an envelope SO_4_^2-^/H^+^ antiporter named SULTR3;1 [110]. In addition, three other members of the same family are candidates for such activity [111], but their chloroplast localization awaits validation. Phylogenetic analyses indicated homologues sequences of the SULTR family in major algae groups [112], but they are not predicted to localize in the chloroplast. In fact, distinct evolutionary paths of sulfate transport systems are proposed for green algae, since, in *C. reinhardtii,* a cyanobacterial-like ABC transport system was reported, whose subunits are coded by the *SulP, SulP2, Sbp,* and *Sabc* genes [113]. This is a 380-kDa holocomplex localized to the envelope membranes and proposed to transport sulfate into the chloroplast. This ABC system has homologues in other green algae as well as in the red alga *C. merolae.*

Iron–sulfur (Fe–S) clusters participate in photosynthesis and other metabolic pathways, but it is not known how they are transported into the chloroplast. SufBCD (sulfur mobilization) is an ABC transporter that was first characterized in *E. coli* and proposed to function in Fe–S cluster biogenesis also in chloroplasts of land plants and algae [114]. In *A. thaliana,* NAP7 is a SufC-like protein, which corresponds to the peripheral ATP-binding subunit of the complex. NAP7 was localized to plastids, and mutant embryos contained abnormal developing plastids with disorganized thylakoid structures, suggesting a role in biogenesis and/or repair of oxidatively damaged Fe–S clusters [115]. It seems that *A. thaliana* plastids also harbor SufA, SufB, SufS, and SufE homologues, implying that plastids probably contain a complete SUF system. No algal homologues of this ABC transporter have been characterized so far. Noteworthy, the ABC-type dominates among *A. thaliana* transporters [116], but only a few homologues sequences are present in the genomes of major algal groups [117], reflecting the relatively simple life of a unicellular organism able to take up nutrients directly from the environment.

### Nitrite transport

Since nitrite assimilation takes place in the chloroplast, transporters are required to supply it into the stroma. The molecular players involved differ completely in land plants from those found so far in algae. The CsNrt1-L is an envelope-located member of the H^+^-dependent oligopeptide transporter family in *A. thaliana*, and was shown to function in nitrite uptake in yeast and chloroplast assays [118]. CLCe was also suggested to play role in nitrite assimilation in *A. thaliana* [119], but other reports found it to be important for Cl^−^ homeostasis and photosynthesis [94, 95]. In the green alga *C. reinhardtii,* the NAR1.1 member of the bacterial formate/nitrite transporter family was shown to mediate nitrite transport into the chloroplast, and to improve nitrate use efficiency for cell growth under low CO_2_ environments [120]. The NAR1.1 has two other chloroplast homologues, NAR1.2 and NAR1.5, and NAR1.2 is a bispecific nitrite and bicarbonate transporter strongly regulated by CO_2_ availability ([121]; see also “Bicarbonate transport”). Such a checkpoint for cross-regulation of carbon–nitrogen metabolism mediated by a transporter is still missing in plants [122]. NAR1 homologues have been found in diatoms [123], but have not yet been investigated in other algal groups.

### Potassium transport

Transport of K^+^ and other cations into the chloroplast is mainly mediated by channels and exchangers driven by the H^+^ gradient across the envelope or thylakoid membranes [73]. Although K^+^ is the major cation in the chloroplast and transport activities have been reported and hypothesized to affect pmf and photosynthesis [124], no algal genes have been thus far identified. In *A. thaliana,* TPK3 was characterized as a thylakoid K^+^ selective channel that lowers the pmf partitioning to ΔΨ by efflux of K^+^ from the thylakoid lumen [125]. The *tpk3* mutants displayed altered photosynthesis, defective thylakoid organization, reduction in growth, and enhanced sensitivity to high light. Three members of the K^+^/H^+^ exchange family (KEA) have been also described in the *A. thaliana* chloroplasts, namely KEA1 and KEA2 in the envelope and KEA3 in thylakoids [126]. They were proposed to play role in K^+^ and pH homeostasis in chloroplasts, which, in turn, are important for chloroplast integrity and optimal photosynthesis [126]. A specific role in photosynthetic acclimation following transitions from high-to-low light was reported for KEA3 [127]. The mechanism behind is that KEA3 imports K^+^ into the lumen in exchange for H^+^ and thus downregulates the pH-dependent NPQ mechanisms to maximize photochemistry. Transport mechanisms to regulate K^+^ and pH homeostasis must also exist in algae to respond to developmental and environmental signals. Homologues of chloroplast KEAs were found in the available sequenced genomes of green algae, red algae, and diatoms but not in glaucophytes [90, 128], whereas TPK3-like sequences were found only in green algae and diatoms [90]. KEAs were also found in secondary endosymbionts such as cryptophytes, implying a function in K^+^ transport across one or more of the four envelope membranes [128].

### Magnesium transport

After K^+^, Mg^2+^ is the second most abundant cation in algae and land plants, since it is a component of chlorophyll, cofactor of enzymes, and is important for thylakoid membrane organization [129, 130]. In *A. thaliana*, one member of the MGT bacterial CorA Mg^2+^ transporter family (also known as MRS2 based on similarity to yeast MRS2) named MRS2-11/MGT10 was localized to the chloroplast envelope [131–133], where it forms large complexes with a composition that may differ from those in bacteria [132]. Initially, a role in Mg^2+^ uptake was demonstrated in yeast complementation experiments as well as using overexpressing mutants [131]. However, based on the elevated Mg^2+^ content of leaves as well as of chloroplasts isolated from *mgt10* mutants as compared to the wild-type plants, a function in the export of Mg^2+^ from the chloroplast was suggested [133]. Mutant analyses also brought evidence for the importance of MGT10 for optimal chloroplast development and photosynthesis [132, 133]. In fact, no mutants with complete loss of MGT10 could be isolated, and it was suggested that the null allele may be lethal at the early stage of embryo development [132]. Two chloroplast homologues were characterized in rice [134] and phylogenetic analyses identified one MRS2-like sequence in the cryptophyte *G. theta* [131]. Such sequences are also recognized in the genome of *C. reinhardtii* [132, 135], but studies are awaiting to investigate their possible location and role in algal chloroplasts.

### Manganese and calcium transport

Photosynthetic water oxidation is performed by a Mn_4_CaO_5_ cluster associated with PSII on the lumenal side of the thylakoid membrane [67]. Ca^2+^ is also a universal signaling molecule [100], and Ca^2+^-sensing proteins (CAS) have been identified in the thylakoid of both *A. thaliana* and *C. reinhardtii* [136–138]. This implies that Mn^2+^ and Ca^2+^ ions are imported into the thylakoid lumen, and indeed, experimental evidence for a biochemical activity of a Ca^2+^/H^+^ exchanger has been reported in pea thylakoids [139].

A member of the H^+^/cation exchanger (CAX) family in *A. thaliana* named PHOTOSYNTHESIS AFFECTED MUTANT71 (PAM71) was localized to the thylakoid membrane, and was shown to function in Mn^2+^ uptake into the thylakoid lumen [140]. Mutants deficient of PAM71 displayed severe growth phenotype due to reduced Mn^2+^ content in PSII and impaired water-oxidizing activity of this complex. Loss of a PAM71 homologue named Conserved in Green Lineage and Diatom 1 (CGLD1) in *C. reinhardtii* mutants resulted in minimal growth and PSII activity, but supplementation of Mn^2+^ restored photosynthesis and growth [140, 141]. Exposure of wild-type cells to high light or peroxide-oxidative stress induced upregulation of the *CGLD1* gene expression, whereas the mutants displayed enhanced sensitivity to these conditions [141]. Remarkably, the mutants are more resistant to singlet oxygen stress, which is attributed to an upregulated expression of several stress-responsive genes [141] and to an increased NPQ [142] by a mechanism that remains to be clarified.

PAM71 also shares homology with yeast and human Ca^2+^/H^+^ antiporters from the CaCA superfamily [143, 144]. Indeed, the same *A. thaliana* protein was characterized as a chloroplast Ca^2+^/H^+^ antiporter (CCHA1) [145]. A paralogue of PAM 71, named PAM71-HL, was localized to the envelope [146], but its function has not yet been studied. PAM71 and PAM71-HL are conserved in green algae such as *C. reinhardtii* and *V. carteri,* and red algae such as *G. sulphuraria*. Since PAM71 participates in Ca^2+^ homeostasis, important roles can be hypothesized in algal physiology, including the activation/deactivation of photoprotective mechanisms [147], high-light response [148], regulation of the ATP availability for CO_2_ fixation [149], and in CCMs [150]. The possibility of a broad divalent cation specificity of AtPAM71 and its algal homologues requires detailed electrophysiological investigations.

### Iron transport

The chloroplast is probably the organelle where iron is the most abundant in the cell, since it is an essential cofactor for the electron transfer chain and catalytic processes such as chlorophyll biosynthesis and protein import [151]. Indeed, iron deficiency has multiple effects on photosynthesis in land plants and algae [152–155]. For its transport into the plant chloroplast, there are several envelope Fe^2+^/H^+^ exchange systems, namely permease in chloroplasts 1 (PIC1) that interacts with the putative metal transport protein NicO, the multiple antibiotic resistance 1, also known as iron regulated 3 (MAR1/IREG3), and the non-intrinsic ABC transporter NAP14/ABCI11 (reviewed in [155]). In addition, ‘yellow stripe 1-like’ (YSLs) were characterized as efflux transporters, and suggested to be involved in iron detoxification of chloroplasts during embryogenesis and senescence [156]. However, Conte et al. [157] questioned the chloroplast localization of YSLs, since they were also found associated with the tonoplast and ER membranes.

The mechanism by which iron is transported into the chloroplast of algae is largely unknown, but homologues of NicO do exist in green algae [158]. In *C. reinhardtii*, expression of NicO increases with decreasing iron concentration but not in zinc or copper deficiency, making NicO a candidate for plastid iron transport in green algae.

Two envelope/thylakoid iron transporters have been characterized in maize and are known as Fe-deficiency-related FDR3 and FDR4 [159, 160]. They are missing in *A. thaliana* but share homology with bacterial FliN and FliP proteins of the secretion system, respectively. ZmFDR4 has homologues in green algae and is induced by iron deficiency in yeast [159]. Despite the essential roles of Fe^2+^ in the chloroplast and the large number of iron transport genes identified in plants, no homologues in other than green algae have been found. Such genes must also exist in, for example, red and brown algae, where iron deficiency was found to reduce chlorophyll content and increase the aggregation of light-harvesting I complexes [161], effects that are also documented in land plants [154].

### Copper transport

Copper is an element required for several chloroplast activities, including the electron carrier protein PC and the antioxidant enzyme superoxide dismutase [70]. Two types of ATP-driven pumps function in tandem to deliver Cu^+^ to the chloroplast stroma and thylakoid lumen in *A. thaliana.* PAA1 and PAA2 (also known as HMA6 and HMA8, respectively) transport Cu^+^ across the envelope and thylakoid membranes, respectively [162, 163]. The envelope-localized HMA1 provides an additional route for import of Cu into the chloroplast, essential under excess light conditions [164]. HMA1 differs from PAA1 in that it can also import other metals/divalent cations (Zn^2+^, Ca^2+^, Cd^2+^ and Co^2+^; [165]). There is some disagreement between studies whether HMA1 can also export divalent cations ([164] and references therein).

HMA1 homologues were found in all major algal groups, whereas PAA1 and PAA2 were present in green algae but not in glaucophytes, red algae, and diatoms, suggesting secondary loss in these taxons [90, 166, 167] . Such a loss may be linked to the absence of PC, since transfer of photosynthetic electrons from cytochrome *b*_6_*f* to PSI is carried out by the iron-containing cytochrome *c*_6_ in glaucophytes, red algae, and diatoms [87, 168]. Interestingly, when copper is limiting in the environment, many green algae replace PC with cytochrome *c*_6_, which, in turn, is replaced back by PC under copper-replete conditions [169]. Homologues of PAA1 and PAA2 were identified in the diatom *T. pseudonana*, but, since they do not possess signal peptides, they are probably involved in export of Cu^+^ to the Golgi compartment [170].

## Chloroplast metabolite transport

To supply the cell and the organism with primary metabolites, a large number of precursors, end products and intermediates have to be transported in and out of the chloroplast. In addition, chloroplasts cannot make ATP at night and must import it from the cytosol. Therefore, they are extensively connected to the cytosol by metabolite transporters that reside in the envelope membranes [85, 171]. Below, we will review the current knowledge about transport in chloroplasts of primary and secondary endosymbionts of ATP, carbohydrates, bicarbonate, organic acids, amino acids, fatty acids, and lipids. Such knowledge is important since these metabolites participate in photosynthetic carbon fixation, photorespiration, biosynthesis of sugars, lipids and proteins, and must cross two-to-four envelope membranes to make a link between the cytosol and the chloroplast stroma. Extensive reviews have been published on metabolite transporters from the plant chloroplasts [84–86], but none so far have been dedicated to algal counterparts.

### ATP transport

There are two structurally and phylogenetically different types of ATP transporters represented in chloroplast membranes, namely the plastidic nucleotide translocators (NTT1 and NTT2) [172], and the thylakoid ATP/ADP carrier (TAAC) [173]. Plant NTTs originate from the obligate intracellular bacteria *Rickettsia* and *Chlamydia*, which get ATP generated by their eukaryotic host [172]. The spinach NTT was shown to work in the same direction, namely supply the stroma with cytosolic ATP in exchange for ADP during the dark period, and the responsible genes were identified in *A. thaliana* (NTT1 and NTT2; [172]). In the same report, reduced amounts of thylakoids were shown in the mutants, suggesting a role of NTTs in supplying ATP for chlorophyll biosynthesis and import of nuclear-encoded proteins, which cannot be compensated by other transport proteins.

NTT homologues were found in the glaucophyte *C. paradoxa*, and the red alga *G. sulphuraria*, indicating an ancient origin for the NTT family [85]. The red alga NTT transports ATP into the stroma in exchange for ADP, i.e., like the plant homologues [174]. Putative NTTs were identified in the diatoms *T. pseudonana* and *P. tricornutum* and were named NTT1 and NTT2 [175]. Both proteins were localized to the envelope and shown to participate in nucleotide supply to plastids, but by distinct mechanisms from each other and from the ATP/ADP exchangers in primary plastids. NTT1 is an H^+^ ATP symporter, whereas NTT2 facilitates the counter-exchange of ATP for (deoxy-)nucleoside triphosphates (ATP/dNTP), thus resembling more those in obligate intracellular bacteria.

As compared to land plants and green algae, diatoms do not generate as much pmf and ATP to maintain the required ATP/NADPH ratio for optimal CO_2_ fixation into biomass [176, 177]. Nevertheless, diatoms are among the most important contributors to the primary production in the ocean [178]. The mechanism behind was unraveled in *P. tricornutum* by Bailleul et al. [179] and involves extensive import of mitochondrial ATP (in exchange for chloroplast NADPH), that must take place across four envelope membranes. Whether NTT1/2 could play role in the import of mitochondrial ATP into the chloroplast to supply the necessary energy for photosynthesis and growth of diatoms remains to be demonstrated.

TAAC belongs to the mitochondrial carrier family and transports ATP in exchange for ADP across the thylakoid membrane [173]. ATP supplied by TAAC is required in the multistep repair of PSII during light stress in *A. thaliana* [180, 181]. TAAC was also localized to the envelope and found to use additional substrates such as phosphoadenosine 5′ phosphosulfate [182]. The envelope-localized protein is known as PAPST1, and is proposed to play role in sulfur metabolism, including the biosynthesis of thiols, glucosinolates, and phytosulfokines. Phylogenetic analyses revealed TAAC/PAPST1 homologues in green algae, but not in red algae, brown algae, and diatoms [183]. This indicates that the gene arose before the divergence of green algae and land plants to fulfill a function specific to these photosynthetic eukaryotes having distinct architecture of the thylakoid membrane [6, 57].

### Carbohydrate transport

Carbohydrate transport across chloroplast envelope membranes is performed in exchange with Pi by plastidic phosphate translocators (pPTs), namely triose-phosphate translocators (TPTs), phosphoenolpyruvate translocators (PPTs), glucose-6-phosphate translocators (GPTs), and xylulose-5-phosphate translocators (XPTs) [171]. The exchange of Pi with phosphorylated compounds guarantees a balance in the Pi content between the stroma and the cytosol and a constant provision of Pi to sustain ATP synthesis [85, 171].

Phylogenetic analyses suggest that pPTs of Archaeplastida evolved from a nucleotide-sugar translocator that resided in the host endomembrane system before the cyanobacterium ancestor was captured [184]. The nucleotide-sugar translocator gene of host origin was duplicated, and after having received the relevant targeting signals evolved into a gene coding for a pPT, providing the host cell with access to the energy produced by the endosymbiont’s photosynthetic machinery. The monophyletic origin of the pPT family reflects its establishment early in plastid evolution, likely at the stage of the ‘proto-alga’ [185]. All three types of pPTs were found in green algae, while only TPTs and PPTs were identified in red algae [85]. Chromalveolates contain several pPTs that originated monophyletically from the TPT clade, whereas PPTs, GPTs and XPTs were apparently lost during secondary endosymbiosis [184]. To date, no typical pPTs have been identified in glaucophytes, but Price et al. [186] identified in the genome of *C. paradoxa* potential hexose-phosphate transporter genes closely related to the UhpC transporters of *Chlamydia*-like bacteria, such genes being also present in red and green algae genomes.

#### Triose-phosphate transport

TPT was the first plant pPT characterized at the molecular level to mediate the exchange of G3P and 3-phosphoglycerate (3-PGA) produced in the chloroplast for cytosolic Pi [187]. In green algae such as *C. reinhardtii*, the upper half of glycolysis (from hexose-phosphate to 3-PGA) is localized inside the chloroplast, while the lower half (from triose-phosphates to pyruvate) takes place in the cytosol [37]. Therefore, TPTs of green algae are expected to accept as substrates G3P, 3-PGA and dihydroxyacetone phosphate (DHAP) with a reversible action, the directionality being determined by the concentrations of NAD(P)H and ATP in the chloroplast and cytosol. Active transport of the metabolites described above was proven in experiments using labelled CO_2_ by several laboratories [188–190]. Although the genes coding for TPTs have been found in the *C. reinhardtii* genome [135], the proteins have not yet been characterized.

In red algae such as *G. sulphuraria*, phylogenetic analysis revealed a candidate orthologue (GsTPT) to the chloroplastic TPT [184]. The GsTPT is able to transport DHAP and G3P but not 3-PGA, most likely representing an adaptation of carbon metabolism in red algae [191]. In contrast to land plants and green algae, red algae do not store starch in the chloroplast. Instead, they produce storage carbohydrates such as floridean starch and floridoside (α-d-galactopyranosyl-1-2′-glycerol) in the cytosol [192]. Therefore, the GsTPT exports G3P from the chloroplast even when present at low stromal concentrations, to fuel the massive demand of carbon in the cytosol [191]. Since GsTPT is unable to transport 3-PGA, its role in the NADPH/ATP shuttle as mentioned for green algae can be excluded [30]. In red algae growing heterotrophically, the GsTPT imports G3P into the plastid and sustains the carbon metabolism and NADPH production, thus by-passing the requirement for a GPT that is functional in plant heterotrophic (non-photosynthetic) plastids [191].

Metabolite exchange across four envelope membranes into secondary plastids becomes more complicated. In the cryptophyte *G. theta*, two TPTs (TPT1 and TPT2) have been reported [193]. As highlighted by assays using isolated complex plastidial membranes, these TPTs are able to catalyze the exchange of Pi with DHAP and phosphoenolpyruvate (PEP), but not with 3-PGA. This feature is reminiscent of the biochemical characteristics of the TPT from the red alga *G. sulfuraria* [191]. Considering that cryptophytes acquired photosynthesis through secondary endosymbiosis of a red alga (Fig. 1), the biochemical properties of their TPTs have not been modified dramatically after the entry of the symbiont into the host cell. The localization of starch granules in the PPC is also consistent with the plastid having originated from a red alga storing floridean starch in the cytosol [194]. Haferkamp et al. [193] showed that night and day paths of sugar metabolism are regulated by differential expression of the two *TPT* genes in *G. theta*. *TPT1* is highly expressed during the night, whereas *TPT2* is mainly expressed in the light. The authors of this study proposed that TPT1 could be localized to the third and/or fourth membrane separating the PPC from the host cell cytoplasm (cER/PPM), where it could export starch degradation products to the cytosol. TPT2 probably resides in the IEM, where it exports triose-phosphates to the PPC to drive starch synthesis in the cytosol.

In the diatom *P. tricornutum*, four putative TPTs were identified in three out of the four plastid membranes [195]. TPT1 was localized in the cER membrane, TPT2 in the PPM, and TPT4a and TPT4b in the IEM, all of them being most likely the product of gene duplications of the red algal endosymbiont transporter gene [195]. These translocators have similar characteristics to their homologues in cryptophytes and apicomplexans, and thus are believed to connect ‘symbiont’ and ‘host’ metabolism by exchange of DHAP and PEP with Pi [195]. The authors of this study also proposed that two TPTs are required in the IEM due to distinct substrate specificities (DHAP and/or PEP) or to separate the activities for export and import of C3 compounds. Five additional putative *TPT* genes were identified in the genome of *P. tricornutum,* but protein localization and characterization have still to be performed [195]. Four genes coding for TPTs were identified in the eustigmatophyte *Nannochloropsis gaditana* (at least one with a plastid-targeting peptide [196]). Based on transcriptomic, lipidomic and metabolomic analyses, the authors proposed that TPTs in this alga control carbon partitioning between organelles, favouring cytoplasm, mitochondria and ER at the expense of the chloroplast, thus promoting lipid accumulation in the cytosol.

#### Phosphoenolpyruvate transport

In plastids of plants performing C3 photosynthesis such as *A. thaliana*, PPTs mediate the uptake of PEP from the cytosol in exchange with Pi [85, 187]. This is required to drive fatty acid biosynthesis and the shikimate pathway for synthesis of aromatic amino acids and of secondary metabolites such as flavonoids and anthocyanins [187]. Lack of PEP import into the stroma of *A. thaliana* mutant for the PPT caused a severe reduction of metabolites derived from the shikimate pathway such as phenylpropanoids [197]. Shen et al. [198] also proposed a role of PPT in a signaling pathway that regulates the epigenetic status of a subset of nuclear genes in *A. thaliana*. In plants performing C4 photosynthesis, PEP is exported by PPT to the cytosol where it serves as a CO_2_ acceptor of the PEP-carboxylase reaction [71]. Shikimate and phenylalanine biosynthetic pathways have evolutionary origins in the endosymbiotic ancestors [199–201]. Therefore, PPTs are expected to be present and function in similar processes in algae, as well. In the red alga *G. sulphuraria*, a candidate PPT was identified (GsPPT), and the recombinant protein was found to have similar properties as the plant orthologue, since it catalyzes the strict counter-exchange of PEP with Pi [184, 191]. The GsPPT is likely required to supply PEP into the stroma for fatty acid biosynthesis and shikimate pathway as in land plants.

#### Glucose-6-phosphate and xylulose-5-phosphate transport

In land plants, GPTs are restricted to heterotrophic plastids, where they import glucose-6-phosphate (Glc6P), G3P and 3-PGA (in counter-exchange with Pi) for starch biosynthesis, fatty acids synthesis and for the oxidative pentose-P pathways (OPPPs) [202]. Evidence for their function in starch biosynthesis comes from land plant experiments showing reduced starch content in seeds with repressed expression of GPT [203]. Shortage of Glc6P as substrate for the OPPPs results in reduced formation of lipid bodies and non-physiological cell death [204]. XPTs are closely related to GPTs and probably derived from the latter by retrotranscription and genome insertion, as suggested by the lack of introns in *XPT* genes [205]. XPTs accept xylulose-5-phosphate (Xul5P), ribulose 5-phosphate, erythrose 4-phosphate and also G3P and 3-PGA in counter-exchange with Pi [199, 206]. The function of XPTs is mainly to provide Xul5P for the OPPPs inside the plastid, especially under conditions of high demand for intermediates of the cycles [199, 205]. Phylogenetic analysis indicated the monophyletic origin of GPTs and XPTs that likely reflects a recent gene duplication event specific to the green lineage [205]. Candidate genes for GPT/XPT orthologues are, however, present in the genome of the red alga *G. sulphuraria* [191]. Remarkably, these proteins have distinct biochemical characteristics from those of green algae, since in experiments using reconstituted membranes, they poorly used 3-PGA and Glc6P as substrates, and instead mediated a Pi/Pi exchange [191]. The physiological substrates of these potential red algal proteins remain unknown, and it appears that GPT/XPT type of transporters may have been lost during secondary endosymbiosis [85].

The genomes of Archaeplastida but not of Chromalveolata also possess sequences homologous to the bacterial UhpC hexose transporter [166, 186]. The UhpC protein from *E. coli* was characterized as a Glc6P/Pi transporter that also acts as a receptor for expression of the sugar-P uptake system [207]. Karkar et al. [166] have localized two putative UhpC transporters from *G. sulphuraria* and *C. merolae* to the chloroplast envelope; however, their function in carbohydrate transport remains to be validated.

### Bicarbonate transport

Biophysical CCM uses energy-dependent active Ci transport to increase intracellular CO_2_ concentration close to the RuBisCO active site [78–80]. CCM was particularly studied in green algae, where Ci species must cross two barriers, the plasma membrane and the chloroplast envelope, to reach the chloroplast stroma, which is believed to be the primary location for the accumulated Ci pool. The chloroplast of the green alga *C. reinhardtii* is able to take up both CO_2_ and HCO_3_^−^ [208]. The expression of several genes for HCO_3_^−^ transporters and CO_2_ gas channels from the plasma membrane and the chloroplast envelope was found modulated in response to changes in external CO_2_ concentration [209, 210]. So far, three Ci transport proteins have been identified in the plasma membrane of *C. reinhardtii*, all considered as limiting-CO_2_-induced (LCI) proteins: HLA3/MRP1 [211], LCI1 [212], and RHP1 [213]. HLA3/MRP1 and LCI1 are HCO_3_^−^ transporters, whereas RHP1 may function as a bidirectional gas channel to provide sufficient CO_2_ for photosynthesis in the absence of a CCM.

The transport of Ci into the chloroplast is performed by proteins belonging to the formate/nitrite transporter family, namely LCIA (also known as NAR1.2) and LCIB, and of the mitochondrial carrier family, namely the low-CO_2_-inducible chloroplast envelope proteins 1 and 2 (CCP1 and CCP2; [214, 215]). To date, members of the LCIA protein family have been identified in the genome of prokaryotes, yeast, green algae such as *C. reinhardtii, V. carteri* and *Chlorella*, diatoms such as *T. pseudonana* and *T. oceanica,* and eustigmatophytes such as *Nannochloropsis gaditana* [216]. LCIA function as a NO_2_^−^/HCO_3_^−^ transporter [121], and has been localized in chloroplast membranes, as evidenced from membrane fractionation experiments [216]. Yamano et al. [217] demonstrated a cooperative uptake of HCO_3_^−^ by LCIA and by the plasma membrane transporter HLA3, and that the stability of HLA3 is dependent on the presence of LCIA.

*LCIB* and three homologous genes in *C. reinhardtii*, *LCIC, LCID,* and *LCIE,* are all responsive to limiting CO_2_, with *LCIB* and *LCIC* among the most abundant transcripts [214]. LCIB orthologues can also be found in other green algae (*Ostreococcus*, *V. carteri*, *Dunaliella*, *Chlorella,* and *Closterium*) and diatom species (*P. tricornutum*, *T. pseudonana,* and *Chaetoceros neogracile*) [218]. For a long time, LCIB was considered as being a Ci transporter, but no transmembrane domain was identified. It was actually found to be a soluble protein either dispersed throughout the chloroplast stroma or concentrated mainly in a region surrounding the pyrenoid [215]. In association with LCIC, it forms a 350-kDa hexameric complex in the region surrounding the pyrenoid that could be involved in trapping the CO_2_ released from the thylakoid lumen via its rehydration into the stromal HCO_3_^−^ pool [218]. This mechanism could potentially involve the stromal CAH6. HCO_3_^−^ is the major species accumulated internally, even though only CO_2_ can be the substrate for RuBisCO. Thus, the CCM also depends on co-localization of CAH6 with or near RuBisCO to catalyze dehydration of HCO_3_^−^ and provide near-saturating CO_2_ concentrations for carboxylation of RuBP.

There is experimental evidence in *C. reinhardtii* for a CCM mechanism involving HCO_3_^−^ transport across the thylakoid membrane into the lumen. Here CAH3 converts HCO_3_^-^ to CO_2_, which, due to the acidic pH in the light, becomes the abundant form in the lumen staying above the environmental levels [219]. Based on localization studies, NAR1.2 was initially proposed as a possible thylakoid HCO_3_^−^ transporter [216, 219]; however, another report could not find evidence for such activity [220]. Most recently, the protein encoded by the *Cia8* gene in *C. reinhardtii* and belonging to the Na^+^ bile acid symporter subfamily was localized to the thylakoid membrane [221]. The authors of this study propose that CIA8 could be the Ci transporter supplying HCO_3_^−^ into the thylakoid lumen with the help of the H^+^ gradient generated in the light. The *cia8* knockout mutant displayed reduced Ci uptake and photosynthetic rates in low CO_2_ conditions, resulting in reduced growth. Such strong phenotype was not observed for *nar1.2* mutants, indicating that CIA8 makes a significant contribution to the CCM in *C. reinhardtii.* CIA8 has a close chloroplast-predicted homologue in *A. thaliana* (*BASS4*), and similar Na^+^/bile acid transporters genes are also present in the marine diatom *P. tricornutum* [221].

The *C. reinhardtii* CCP1 and CCP2 are chloroplast envelope-localized proteins and belong to a family of carriers with broad substrate specificity [214, 215]. CCP1 and CCP2 are both strongly upregulated in low CO_2_ conditions [222]. However, results brought by RNAi knockdown experiments raised doubts about their role in Ci transport since the mutants exhibited Ci uptake and photosynthesis similar to wild-type cells [223]. The authors suggested that these proteins could be involved in the transport of metabolic intermediates important in acclimation to low CO_2_. The existence of Ci transport systems that would compensate this loss may also explain the obtained results.

Ycf10 has also been identified as a Ci transport candidate in *C. reinhardtii* [214]. It displays a sequence homology with the plastid-encoded CemA protein from land plants and the cyanobacterial PxcA protein [224]. To date, it is still not clear how Ycf10 functions in *C. reinhardtii* chloroplast Ci transport, but a role close to that of PxcA is likely, namely in the light-induced H^+^ extrusion from the chloroplast stroma [214]. This may be required to maintain the alkaline stromal pH for functional CCM in chloroplasts.

The LCI11 and MITC11 proteins have also been suggested to be involved in the CCM, since their transcripts were found strongly upregulated during growth under low CO_2_ conditions in the green alga *C. reinhardtii* [209] and the diatom *T. pseudonana* [225]. LCI11 is predicted as a chloroplast member of the bestrophin (Best) family, which can transport organic and inorganic anions including HCO_3_^−^ [226]. LCI11 shares weak similarity to the *A. thaliana* Cl^−^ channel VCCN1 [96], suggesting that they may have a common origin but diverged to fulfill different functions in algae and land plants. MITC11 shows homology to mitochondrial carriers, and localization predictions place it as a chloroplast protein [80]. Localization and functional studies for these two transporters have not yet been performed in algae.

### Organic acid and amino acid transport

Biochemical CCM in algae depends on exchange of OAA and malate between the chloroplast and the cytosol [72]. Interestingly, *Best1* and *Best2* genes were found upregulated in low CO_2_ conditions in the diatom *T. pseudonana*. They are homologues of *C. reinhardtii* LCI11 and are suggested to transport OAA into the chloroplast [225], which could be in line with the previous observations about Bests being permeable to organic anions [227]. In *C. reinhardtii*, chloroplasts and mitochondria exchange OAA and malate via the envelope LCI20 and the mitochondrial OMT [37]. The *C. reinhardtii* LCI20 protein is orthologous to the *A. thaliana* envelope 2-oxoglutarate/malate translocator (DiT1), and was found in the chloroplast proteome [3].

As mentioned earlier, the presence of an elevated concentration of oxygen in the proximity of RuBisCO results in oxygenase activity and photorespiration at the expense of carbon fixation. The photorespiratory metabolism is distributed in several specialized compartments (mitochondria, chloroplasts and peroxisomes), and translocators facilitate the metabolite flux through the cycle. Eisenhut et al. [84] performed a phylogenetic analysis of known dicarboxylate transporters, DiT1, DiT2.1 and the plastidial glycolate/glycerate transporter PLGG1 from *A. thaliana*. Homologues PLGG1 sequences could be found in all algal groups and cyanobacteria, indicating that it had a cyanobiont origin. Green algae are distinct in the pathway as they do not have peroxisomes and perform the glycolate oxidation using a glycolate dehydrogenase in mitochondria. This difference implies that the mitochondrion needs a transporter comparable to the plastidic PLGG1, facilitating glycolate import and glycerate export. Indeed, homologues PLGG1 sequences found in *C. reinhardtii* and *V. carteri* had dual predicted location to mitochondria and chloroplasts. DiT1 and DiT2.1 have homologues in green algae but not in red algae or glaucophytes, implying that they have been acquired via horizontal transfer from *Chlamydia* or that they have been lost in the former [84].

Amino acids are the building blocks of proteins, and in land plants and algae, they are synthesized in plastids. They can be used within plastids or are exported to the cytosol and mitochondria. Very little is known about the transport of amino acids into or out of chloroplasts. DiT2.1 is the only characterized chloroplast amino acid transporter in *A. thaliana* [228]. It mediates the exchange of glutamate for malate and can also use the amino acid aspartate as a substrate. Members of the preprotein and amino acid transporter family in *A. thaliana*, PRAT1 and PRAT2, were localized to the chloroplast envelope and proposed to mediate export of amino acids to the cytosol [229, 230]. Experimental evidence is available for the function of PRATs in protein import in plants, and homologues were found in three green algae, namely *C. reinhardtii*, *V. carteri* and *Ostreococcus lucimarinus* [231].

### Fatty acid and lipid transport

Fatty acids (FA) and lipids are not only used for membrane building but also for development and growth of cells. Indeed, some FA and lipids cannot be produced by human cells—the so-called essential lipids, and must be acquired through diet [232]. In land plants and algae, FA are synthesized in plastids, exported to the ER for modifications and lipid assembly, and ultimately distributed within the cell [8, 233]. Some lipids are reimported for plastid-specific lipid assembly [8, 234]. Green algae such as *C. reinhardtii* have a similar lipid composition of chloroplast membranes to land plants [235].

Because lipophilic molecules such as FA cannot freely move in an aqueous environment, several modes of transport have been identified. These include membrane contact sites, diffusion, flip transfer, vesicular trafficking and protein-mediated transport. In the land plant *A. thaliana,* several ABC FA/lipid transporters have been described, but none of them were from the chloroplast [236]. Fatty Acid Export 1 (FAX1) was localized to the chloroplast envelope and proposed to transport FA from the stroma to the inter-envelope space [237]. From there, FA could move to the cytosol thanks to the vectorial acylation transport mediated by long-chain acyl-CoA synthetase 9 (LACS9). FAX1 belongs to a 7-member family in *A. thaliana* of which FAX2, FAX3, and FAX4 have been also predicted as plastid proteins, and could compensate for FAX1 loss of function [238]. Phylogenetic analyses indicated that plastid FAX homologues are restricted to land plants and green algae [237]; however, no algal FAXs have been experimentally characterized.

For the assembly of chloroplast-specific lipids, they should be reimported into plastids [8]. TRIGALACTOSYLDIACYLGLYCEROL1-3 (TGD1-3) is an ABC transport complex-mediating lipid transfer from the inter-envelope space to the chloroplast stroma [239, 240]. In this complex, TGD1 is an inner envelope intrinsic permease transporting the phosphatidic acids bound to TGD2 into the inter-envelope space. The energy for the transport is provided by the stroma-located ATPase subunit TGD3 [238]. A TGD5 protein was identified in *A. thaliana* and hypothesized to facilitate lipid transfer from the outer envelope to the inner envelope by bridging phosphatidic acid-binding β-barrel lipid transfer protein TGD4 with the TGD1-3 complex via protein–protein interactions [241]. The exact nature of the lipid species transported by the TGD complex in plants is not fully elucidated. The *C. reinhardtii* genome encodes putative plant orthologues of the chloroplast TGD1-3 complex required for the ER-to-chloroplast lipid trafficking [242]. The same study localized the CrTGD2 to the inner envelope and reported on the reduced viability of a *tgd2* mutant due to altered galactoglycerolipid metabolism. Since TGD4 and TGD5 are apparently absent in the *C. reinhardtii* genome, the authors proposed that the TGD1-3 complex may also perform the transfer at the outer envelope membrane.

It is established that in nutrient (nitrogen and phosphorus) deprivation conditions, algae accumulate lipids, but the control points that direct fixed carbon into lipid accumulation have not been fully elucidated (green algae: [243], diatoms: [244]). The biophysical CCM mechanism involving HCO_3_^−^ transport is the preferred route for CO_2_ fixation during nutrient deprivation [245]. As documented above, lipid biosynthesis and transport have been studied much less in algae than in plants, although such knowledge is required to engineer strains for biotechnological applications [246–249].

## Strategies for identification of missing chloroplast transporters in algae

Although the chloroplast hosts photosynthesis and multiple biosynthetic pathways vital for the plant and algal cells, the transporters mediating the exchange of ions and metabolites are mostly unknown even in *A. thaliana* [83, 250]. The summary presented in Supplemental Table S1 and illustrated in Fig. 3 clearly indicates the overwhelming number of missing algal transporters for both ions and metabolites, whose identification would greatly impact our understanding of the chloroplast homeostasis and metabolism. Bioinformatic analyses combined with experimental strategies have been used for identification and characterization of many chloroplast transporters from *A. thaliana* [88, 251]. Heterologous expression provided insights into the type of substrate, whereas analyses of mutants revealed their physiological roles. Mass spectrometry-based proteomics revealed the initial information about localization [146, 252, 253] and were followed in many cases by more targeted approaches using fluorescent markers (for reviews, see [81–86]). Similar strategies could be used to identify and characterize missing chloroplast transport proteins encoded by genes in the various algal models (Supplemental Table S1). In addition, co-expression analyses of the known transporters in algae could aid to unravel new genes for transporters involved in common processes in the chloroplast [254]. Prediction tools for transmembrane topology, localization and phylogeny similar to those available for plants at ARAMEMNON [255] could be employed to mine the information in their sequences.

A phylogenomic and network analysis approach was used to identify homologues in algae of validated metabolite transporters from the chloroplast envelope of *A. thaliana* [166]. Bioinformatic and protein localization in tobacco chloroplasts could show plastid targeting for two red algal putative UhpC transporters. This study also showed that more than half of the envelope transporter genes are of eukaryotic origin and that the captured cyanobacterium made a relatively minor contribution to the process. Similar analyses could also be performed using transporter gene sequences from cyanobacteria and non-photosynthetic bacteria. For instance, a novel and specific pyruvate/H^+^ symporter has been reported in *E. coli* [256]. Such a transporter could have been acquired through horizontal gene transfer, enabling the use of pyruvate as a carbon source for the growth and survival of *E. coli*.

Some collections of algal mutants are available (e.g., Chlamydomonas Resource Center, http://chlamycollection.org/), and in addition, the new technique of genome editing with the help of CRISP/CAS9 system could be employed to generate knockout and knockdown mutants for algal genes absent in collections [257]. Using algal mutants defective in the activity of putative transport proteins could allow validating their function and physiological role as it has been done successfully for plant counterparts from the chloroplast envelope [86] and thylakoids [88]. Indeed, many mutants of chloroplast transporters displayed altered photosynthesis in *A. thaliana* and *C. reinhardtii*, since they occupy key positions in the pathway exchanging ions and metabolites across chloroplast membranes (for reviews, see [81, 83, 98, 258, 259]).

## Conclusions and perspectives

In this review, we have provided an overview of the current knowledge about ion and metabolite transport in the chloroplast of algae. Some of the reviewed studies reported on the localization and transport function, whereas most studies predicted the existence of genes that are homologues to those of known plant transporters. Most transporter genes were identified in the green alga *C. reinhardtii*, but some were found in models for red algae, diatoms, glaucophytes or cryptophytes. Most identified chloroplast transporters reside in the envelope and participate in carbon acquisition and metabolism. Only a few are located in the thylakoid membrane and play role in ion transport. The strategies used to characterize chloroplast transporters from plants could inspire future work in algae.

Algae represent a potentially non-expensive, scalable, CO_2_-fixing, solar-powered source of diverse natural products such as lipids, pigments and proteins, that are synthesized mainly in the chloroplast (e.g., [4, 8]). One recent research development is synthetic biology, i.e., ‘the deliberate (re) design and construction of novel biological and biologically-based systems to perform new functions for useful purpose, that draws on principles elucidated from biology and engineering’ [260]. Through genetic engineering, synthetic biology can transform algae and their chloroplasts in cell factories for the production of exotic compounds such as vaccines and antibiotics [247]. In this respect, the fact that the chloroplast genome is of prokaryotic origin offers many advantages because it is easily amenable. Despite of this enormous potential, the development of algal biotechnologies remains fragile, especially because of lack in a deep knowledge in the biochemistry, physiology, and stress responses of the algal cell activity [28], that also impact the economic viability of these technologies [2].

A better knowledge about ion and metabolite chloroplast transporters would help in the recovery of compounds from algal cells within downstream processing. This processing aims to disrupt the physical and mechanical cell barriers preventing the recovery of interesting compounds. Several chemical and physical methods have been proposed to help the recovery process [2, 261], but they have been thus far only applied to small-scale tests [261]. The manipulation of intracellular compound circuits in intact algae using chloroplast transporters would certainly enable new opportunities in algal research and in the use of algae as photoautotrophic tools for biotechnological applications. At the same time, such studies would serve to advance our understanding of biological barriers, a goal of central significance in the life sciences, agricultural and medical research.

## Electronic supplementary material

Below is the link to the electronic supplementary material.

Supplementary material 1 (PDF 251 kb)
