# Supplementary material for: Ion and metabolite transport in the chloroplast of algae: lessons from land plants
Source: Cell Mol Life Sci. 2018 Mar 14;75(12):2153–76. doi: 10.1007/s00018-018-2793-0 (PMC5948301; doi:10.1007/s00018-018-2793-0)
Supplement: Supplementary file 1 — Supplementary material 1 (PDF 251 kb) [file 18_2018_2793_MOESM1_ESM.pdf]

Supplemental Table S1. Ion and metabolite transport proteins in the chloroplast from algae and land plants.
+, gene present; –, gene absent; n.d., not determined. The source of the data is indicated in the last column (reference numbers as in the text).

**Type of transport**	**Substrate**	**Land plant**	**Algal group and models**					**Chloroplast localization**	**References**
			Green algae	Red algae	Diatoms	Glaucophytes	Cryptophytes		
		Arabidopsis thaliana, Zea mays	*Chlamydomonas reinhardtii, Volvox carteri*	*Galdiera sulphuraria, Cyanidioschyzon merolae*	*Thalassiosira pseudonana, Phaeodactylum tricornutum*	*Cyanophora paradoxa*	*Guillardia theta*		
**Chloride transport**	Cl^-	AtVCCN1, AtVCCN2	+	+	+	–	–	Thylakoids	[96, 97]
	Cl^-	AtCLCe	+	–	+	–	+	Thylakoids	[90, 94, 95]
	Cl^-	AtMSL2, 3	MSC1	+	+	+	+	Envelope	[100-102]
**Phosphate transport**	Pi, H^+, Na^+	AtPHT4;1	+	+	–	–	–	Thylakoids	[90, 104, 105]
	Pi, H^+, ascorbate	AtPHT4;4	+	+	–	–	–	Envelope	[90, 104, 107]
	Pi, H^+	AtPHT2;1	Only charophytes	–	–	–	–	Envelope	[103, 109]
**Sulfur transport**	SO_4^{2-}	AtSULTR3;1	SulP, SulP2, Sbp, Sabc	+	–	–	–	Envelope	[113]
	Fe-S	AtNAP7, SufABSE	SufBCD	+	–	+	–	Envelope	[114, 115, 117]
**Nitrite transport**	NO_2^-	CsNtrt1-L	NAR1.1	n.d.	+	n.d.	n.d.	Envelope	[120, 121, 123]
**Potassium transport**	K^+	AtTPK3	–	–	+	–	–	Thylakoids	[90, 125]
	K^+, H^+	AtKEA1, 2	+	+	–	–	+	Envelope	[90, 126, 128]
		AtKEA3						Thylakoids	[90, 127, 128]

**Magnesium transport**	Mg^{2+}	AtMGT10	+	n.d.	n.d.	n.d.	+	Envelope	[131-135]
**Manganese and calcium transport**	Mn^{2+}, Ca^{2+}	AtPAM71/CCHA1	CGLD1	+	–	–	–	Thylakoids	[140-142, 145]
		AtPAM71-HL	PAM71-HL					Envelope	[146]
**Iron transport**	Fe^{2+}	AtPIC1, AtNicO	+	n.d.	n.d.	n.d.	n.d.	Envelope, thylakoids	[155, 158]
	Fe^{2+}	ZmFDR3,4	+	n.d.	n.d.	n.d.	n.d.	Envelope, thylakoids	[159, 160]
**Copper transport**	Cu^{+}	AtHMA6/PAA1	+	–	+, no cTP	–	n.d.	Envelope	[90, 162, 167, 170]
	Cu^{+}	AtHMA8/PAA2	CrHMA2-5	–	+, no cTP	–	n.d.	Thylakoids	[90, 162, 163, 167, 170]
	Cu^{+}, Ca^{2+}, Zn^{2+}, Cd^{2+}, Co^{2+}	AtHMA1	CrHMA1	+	+	+	n.d.	Envelope	[164, 166, 167]
**ATP transport**	ATP, ADP	AtNTT1, 2	–	GsNTT	–	CpNTT	–	Envelope	[85, 172, 174]
	H^{+}, ATP	–	–	–	PtNTT1, TpNTT1	–	–	n.d.	[175]
	ATP, dNTP	–	–	–	PtNTT2, TpNTT2	–	–	n.d.	[175]
	ATP, ADP, PAPS	AtTAAC/PAPST	+	–	–	–	–	Thylakoids, envelope	[173, 182, 183]
**Triose-phosphate transport**	G3P, DHAP, 3-PGA, Pi	AtTPT	+	GsTPT	n.d.	–	n.d.	Envelope	[37, 85, 187, 191]
	DHAP, PEP, Pi	–	–	–	PtTPT1, 2, 4a, 4b	–	GtTPT1, 2	Envelope: cER, PPM, IEM	[193, 195]
**Phosphoenolpyruvate transport**	PEP, Pi	AtPPT	+	GsPPT	n.d.	n.d.	n.d.	Envelope	[85, 184, 187, 191]
**Glucose-6-phosphate**	Glc6P, G3P, 3-PGA, Pi	AtGPT	+	+, distinct substrate	–	–	–	Envelope	[85, 191]

**transport**	Glc6P?	–	+	GsUhpC, CmUhpC	–	CpUhpC1, 2	–	Envelope	[166, 186]
**Xylulose-5-phosphate transport**	Xul5P, G3P, 3-PGA, Pi	AtXPT	+	+, distinct substrate	–	–	–	Envelope	[85, 191, 199, 206]
**Bicarbonate transport**	HCO_3^-, NO_2^-	–	NAR1.2/LCIA	n.d.	+	n.d.	n.d.	Envelope	[216, 217]
	HCO_3^-	AtBASS4	CIA8	n.d.	+	n.d.	n.d.	Thylakoid	[221]
	HCO_3^-?	–	CCP1, CCP2	n.d.	n.d.	n.d.	n.d.	Envelope	[214, 215]
	HCO_3^-?	n.d.	MITC11	n.d.	+	n.d.	n.d.	Chloroplast predicted	[80, 209, 225]
	OAA, HCO_3^-?	n.d.	LCI11	n.d.	TpBest1, 2	n.d.	n.d.	Chloroplast predicted	[209, 225, 226]
	H^+, ?	Cem	YCF10	n.d.	n.d.	n.d.	n.d.	Envelope	[214]
**Organic acid and amino acid transport**	OAA, malate	AtDiT1	LCI20	n.d.	n.d.	n.d.	n.d.	Envelope	[3, 37]
	Glycolate, glycerate	AtPLGG1	+	+	+	n.d.	n.d.	Chloroplast predicted	[84]
	Glutamate, aspartate, malate	AtDiT2.1	+	–	n.d.	n.d.	n.d.	Envelope	[84, 228]
	Amino acids	AtPRAT	+	n.d.	n.d.	n.d.	n.d.	Envelope	[229-231]
**Fatty acid and lipid transport**	Fatty acids	AtFAX1-4	+	–	–	–	–	Envelope	[237, 245]
	Triacylglycerides	AtTGD1-3	CrTGD2	n.d.	n.d.	n.d.	n.d.	Envelope	[239, 242]
